# Enhancement of Chromium (VI) Reduction in Microcosms Amended with Lactate or Yeast Extract: A Laboratory-Scale Study

**DOI:** 10.3390/ijerph17030704

**Published:** 2020-01-21

**Authors:** Valeria Ancona, Claudia Campanale, Marina Tumolo, Domenico De Paola, Claudio Ardito, Angela Volpe, Vito Felice Uricchio

**Affiliations:** 1 Water Research Institute-Italian National Research Council (IRSA-CNR), 70132 Bari, BA, Italy; claudia.campanale@ba.irsa.cnr.it (C.C.); marina.tumolo@ba.irsa.cnr.it (M.T.); claudio.ardito@ba.irsa.cnr.it (C.A.); angela.volpe@ba.irsa.cnr.it (A.V.); vito.uricchio@ba.irsa.cnr.it (V.F.U.); 2 Institute of Biosciences and Bioresources, Italian National Research Council (IBBR-CNR), 70126 Bari, Italy; domenico.depaola@ibbr.cnr.it

**Keywords:** hexavalent chromium, yeast extract, lactate, microcosms

## Abstract

A laboratory-scale study was carried out to evaluate the groundwater bioremediation potential of hexavalent chromium (Cr(VI)), taking into account the chromate pollution of an industrial site located in Southern Italy (Apulia Region). The reduction of Cr(VI) was studied on laboratory microcosms, set up in different experimental conditions, namely: ABIO (soil and water sterilized), BIO (soil and water not sterilized), LATT (with the addition of lactate), and YE (with the addition of yeast extract). Control test lines, set up by using sterilized matrices and amendments, were employed to assess the occurrence of the pollutant reduction via chemical processes. By combining molecular (microbial abundance, specific chromate reductase genes (ChR) and the *Shewanella oinedensis* bacterial strain) with chemical analyses of chromium (VI and III) in the matrices (water and soil) of each microcosm, it was possible to investigate the response of microbial populations to different experimental conditions, and therefore, to assess their bioremediation capability in promoting Cr(VI) reduction. The overall results achieved within this work evidenced the key role of amendments (lactate and yeast extract) in enhancing the biological reduction of hexavalent chromium in the contaminated aqueous phase of laboratory microcosms. The highest value of Cr(VI) removal (99.47%) was obtained in the YE amended microcosms at seven days.

## 1. Introduction

The main natural source of chromium in the environment is the erosive process of rocks containing chromite, generally ferric chromite, a mineral in which chromium is present in the oxidative state of +3, hereinafter Cr(III) or trivalent chromium. The principal Cr(III) reaction in water is the formation of chromium hydroxides and mixed iron chromium hydroxide (Cr(OH)_3_ and CrFe(OH)_6_), which have a low solubility and tend to precipitate under neutral to basic aqueous solutions [1]. By contrast, the other form of chromium that is stable in a natural environment, the hexavalent chromium, or Cr(VI), is water soluble at any pH and is 100-fold more toxic than Cr(III) [2]. The toxic action of Cr(VI) is due to its ability to easily penetrate cellular membranes. Cell membrane damages caused by oxidative stress induced by Cr(VI) have also been extensively reported, both in eukaryotic and prokaryotic cells [2,3,4].

While there are natural sources of chromium in the environment, the majority of Cr(VI) inputs are derived from industrial processes, including metal plating, leather tanning, synthesis of paint pigments and dyes, and stainless-steel production [5]. As a result of the improper disposal of waste and wastewater from these industries, Cr(VI) enters ecosystems [6].

Some microorganisms possess mechanisms that enable them to reduce the toxic chromium Cr(VI) to the less toxic trivalent state Cr(III), either as a survival mechanism aimed at reducing the toxicity around the cell, or as a means of deriving metabolic energy for cell growth [7]. In several chromium-resistant bacteria, the ChrR gene codes for a soluble chromate reductase (NADH-dependent), involved in the cytoplasmatic reduction of chromium. Another strategy is the extracellular Cr(VI) reduction, as reported for microorganisms such as *Shewanella oneidensis*. This Gram-negative bacterium, widespread in several environments, can grow both aerobically or anaerobically, and it is considered an important model organism for studies on bioremediation [8,9,10].

Heavy metal pollution represents a serious environmental problem because of its implications on human health and ecosystems. This fact has led to increased interest in developing biotechnology approaches for bioremediation. Particularly, over recent decades, among various approaches, the bioremediation of hexavalent chromium using whole microorganisms or the sole enzymes has attracted more attention because of its relatively cost-effective, sustainable, and eco-friendly characteristics [11]. Bioremediation represents a valid alternative to the chemical methods that involve many reducing agents such as Fe(0), Fe(II), sulfide, and organic C-based materials, particularly as these methods are ineffective at the lower concentrations of Cr(VI) present in large volumes of wastewater, and could generate secondary pollution [12].

However, the potential for the biological treatment of Cr(VI) contaminated waste is limited, because a majority of these bacteria cannot tolerate high concentrations of chromate [13]. Furthermore, most known species capable of chromate reduction are heterotrophic, and need extra nutrition addition during the reduction process, especially in an oligotrophic groundwater environment [11].

The bioremediation of hexavalent chromium by the pure cultures of microorganisms isolated from the contaminated matrix has been reported in numerous studies. Cheng and Li [14] found that bacteria belonging to the genus Bacillus, isolated from soils in an iron mineral area, could remove 100% of Cr(VI) at a concentration of 10 mg/L in 24 h. Bharagava and Mishra [15] reported the case of a bacterium isolated from a tannery wastewater, identified as *Cellulosimicrobium* sp., capable of reducing 99.33% and 96.98% of Cr(VI) at 50 and 100 mg/L in 24 and 96 h, respectively. However, almost no single strain used for in situ bioremediation intervention can compete with the native microbial community. Thus, native microbial communities play a dominant role in the successful outcome [16].

In order to study and evaluate the capabilities of natural microorganisms in favoring the bio-reduction of Cr(VI), laboratory microcosms can represent useful ecosystem models. Microcosms consist of a portion of environmental matrices (e.g., soil, water, and sediment)—containing natural biotic communities—in which temperature, light, and humidity are maintained under control so as to reproduce field conditions. Also, by laboratory microcosm studies, it is possible to assess the influence of amendment addition in enhancing the bioremediation processes [17].

In this research, a series of microcosm experiments have been conducted in order to study the Cr(VI) bioremediation capabilities of the natural microbial communities in a specific aquifer of Southern Italy. On details, the tests were setup in reactors (microcosms), kept under controlled conditions, with the aim of reproducing, at laboratory scale, the natural conditions observed in a real aquifer, polluted by Cr(VI), located in an industrial area of Barletta Municipality [18], and testing the bioremediation potential of hexavalent chromium.

The main goal of the work was to assess the role of different amendments (lactate and yeast extract) in the Cr(VI) reduction process, evaluating their potential as electron donors and as an additional carbon source to the development of autochthonous microorganisms. The response of a microbial population to different experimental conditions has been investigated in terms of microbial growth and hexavalent chromium removal by combining molecular and chemical analyses. In addition, an attempt to identify the genes or microorganisms naturally occurring in the examined groundwater, and possibly involved in chromate bioreduction, has been carried out.

## 2. Materials and Methods 

### 2.1. Matrices 

The choice of matrices (soil and groundwater) was done in order to simulate, as much as possible, the environmental conditions observed in an aquifer contaminated by Cr(VI), located in an industrial area of Barletta Municipality (Southern Italy) [18].Thus, limestone, representative of the saturated zone aquifer of the case study, was used as solid matrix; while the groundwater was taken from a piezometer located inside the Water Research Institute (IRSA-CNR) area, in the industrial zone of the Bari Municipality.

### 2.2. Amendments 

The use of two different types of amendments (lactate and yeast extract), considered suitable for “stimulating” the microbial communities naturally present in the matrices, was adopted to enhance the Cr(VI) reduction processes.

Lactate was chosen because of its capacities to stimulate the Cr(VI) reduction ability of some microorganisms, and to act as an efficient electrons’ donor, as previously reported by many authors [13,19,20].

On the other hand, yeast extract was selected as C sources to accelerate the reduction process of Cr(VI) to Cr(III), as observed in situ by Cifuentes et al. [21].

### 2.3. Experimental Design and Microcosms Setup 

The preparation of the microcosms was carried out by adding a solid matrix (limestone—S) and a liquid matrix (groundwater—GW), inside of each reactor (polyethylene, PE, bottle). The employed GW was previously artificially spiked with a concentration of Cr(VI) equal to 1000 µg/L. In order to recreate an environment that simulates a saturated aquifer, the ratio between S and GW, adopted in the reactor, was 1:4, according to Matteucci et al. [22]; therefore, 50 g of limestone and 200 mL of GW were introduced to each microcosm. An amount of 200 mg/L of yeast extract or 3 mM of lactate was added to the reactors, in accordance with Volpe et al. [23] and Matteucci et al. [22], respectively.

Overall, in order to select the optimal circumstances for the reduction of Cr(VI), four different experimental conditions were set up (Table 1). In each soil and water sterilized (ABIO) reactor, 1 mL of sodium azide (final concentration 1 g/L) was added to prevent any bacterial proliferation over time.

A total number of 60 reactors were prepared in previously sterilized 250 mL PE bottles for investigating the four experimental conditions in triplicate, at five times of incubation (4, 7, 11, 14, and 28 days), which correspond to T1, T2, T3, T4, and T5 investigation times, respectively.

Finally, all of the microcosms were incubated in the dark at 24 °C, and were manually shaken every day in order to accelerate the biological processes of oxygen consumption by the heterotrophic population of microorganisms.

### 2.4. Controls 

A first line of control tests (a total of four reactors) was set up with the same composition as the experimental conditions, but with the addition of 1 mg/L of the redox resazurin indicator, in order to detect the possible changes of the electrochemical potential within each experimental condition.

Resazurin is an indicator whose colour varies according to the redox potential. The blue colour indicates an aerobic condition characterized by a positive redox potential; this colour gradually becomes pink first and then transparent, when anaerobiosis conditions are reached and the redox potential becomes negative.

A second line of control tests was setup in order to investigate whether a chemical reduction of Cr(VI) is associated exclusively to the role of the two investigated amendments as a source of electron donators. To this aim, two experimental conditions were set up (with the addition of lactate (LATT) control and with the addition of yeast extract (YE) control) with autoclaved matrices and autoclaved amendments, for a total of six reactors—three for each amendment. Moreover, sodium azide was also added as a biocide agent, at the same concentration used for the ABIO reactors (§ 2.3).

### 2.5. Chemical Analysis 

The residual chromium concentration in supernatant was determined by a colorimetric assay using S-diphenylcarbazide (DPC), a highly specific complexing agent for Cr(VI) [24].

Specifically, a 25 mL aliquot of a supernatant solution taken from the microcosm was added to 1 mL of 2.5 M H_2_SO_4_ and 1 mL of difenilcarbazide 0.5%.

The absorbance was read after 10 min at 540 nm with a UV-VIS spectrophotometer (Cary 60-UV-VIS, Agilent), using a cell with optical pathlengths of 10 cm to enhance the spectrophotometric detection and provide a suitable sensitivity, achieving a limit of detection as low as of 1 µg/L.

The samples’ absorbance was compared to a standard curve of potassium dichromate prepared in a double range of concentration (low and high range) in order to ensure the results were as accurate as possible.

Inductively coupled plasma-mass spectrometry (ICP-MS; Agilent 7700 Series, Agilent Technologies, USA) was used to measure the total aqueous Cr concentrations in the supernatant at the end of the experiment (T5). The concentration of Cr(III) was then calculated as the difference between the total aqueous Cr and Cr(VI) in solution.

To measure the amount of Cr(VI) reduction products, precipitated on the solid matrix, 500 mg of sample from LATT and YE reactors at T5, were acid microwave digested by HCl and HNO_3_ 3:1 for 15 min at 200 °C and then measured by ICP-MS.

The removal percentage (R%) of the pollutant was calculated using the following equation:

(1) $$R(\%)=\frac{\left(\mathrm{Ci}-\mathrm{Ce}\right)\times 100}{\mathrm{Ci}}$$

where C_i_ is the initial concentration of chromium in the aqueous solution, and C_e_ is the Cr(VI) concentration observed at fixed incubation times.

### 2.6. Molecular Analysis 

#### 2.6.1. Evaluation of Microbial Abundance

Genomic DNA was extracted from the groundwater and limestone. employed to setup the microcosm tests using a DNeasy PowerSoil kit (Qiagen, Germantown, MD). Particularly, in order to extract the DNA from the groundwater, one sample litre was filtered through a 0.22 µm membrane, which was treated as the solid matrix. For the limestone, genomic DNA was extracted from 0.250 g, according to the manufacturer’s instruction.

In addition, DNA was extracted from the aqueous phase (50 mL) of each microcosm at five different times of investigation, as previously described (§2.3).

The integrity of the extracted DNA was checked on 1% agarose gel through electrophoresis. The DNA concentration was also quantified by spectrofluorimetric assays using Qubit 3.0 (Thermo Fisher Scientific, MA, USA).

#### 2.6.2. PCR Assays

Genomic DNA from the groundwater and limestone were applied as a template for the PCR reactions (Table 2) using a TProfessional Thermocycler (Biometra GmbH, Germany).

We used the primer set Eub338/Eub518 for the amplification of the 16S rRNA gene [25], with the dual aim of assessing the extracts’ suitability for downstream PCR applications and evaluating the bacterial presence in each sample.

The 20-µL reaction mixtures contained 0.3 µM of each primer, 10 µL of HotStarTaq^®^ master mix (Qiagen, Germantown, MD), 2 µL of DNA extract, and RNase-free water to complete the 20 µL volume. The conditions for the amplification of the 16S rRNA gene were as follows: 5 min at 95 °C for enzyme activation, followed by 30 cycles of 30 s at 94 °C, 30 s at 54 °C, 30 s 72 °C for the denaturation, annealing, and extension steps, respectively, and a final elongation step of 10 min at 72 °C.

In addition, we checked for two main ways of reducing Cr(VI), namely, the intracellular and extracellular ones. We used two primer sets, ChRF/ChrR and 640F/815R, to detect the intracellular chromate reductase gene (chrR gene) and the V3–V4 hypervariable region of the 16S rRNA gene of *Shewanella oneidensis*, a model organism for the extracellular chromium reduction, respectively [26,27]. PCR amplifications were performed in a total reaction volume of 20 µL, containing the following: 0.3 µM of each primer, 10 µL of HotStarTaq^®^ master mix, 5 µL of DNA extract, and 3.5 µL of RNase-free water. PCR amplification was carried out in a thermocycler subjected to denaturation at 95 °C for 5 min, followed by 35 cycles at 94 °C for 30 s, 50 °C for 30 s, 72 °C for 30 s, and a final elongation step at 72 °C for 10 min.

The PCR products were separated on 1.5% agarose gels and were visualized with UV light.

#### 2.6.3. Assessment of Shewanella Oneidensis in Amended Microcosms

Further investigations were conducted on *Shewanella oneidensis* because of its relevance as a model organism for studies on extracellular chromate reduction.

Thus, quantitative PCR assays (qPCR) were conducted on amended microcosm tests using the primer sets 640F/815R and Eub338/Eub518, in order to measure the abundance of *Shewanella oneidensis* during the experiment, in comparison with the total amount of bacteria, until 28 days.

qPCR analyses were performed on a Rotor-Gene Q (Qiagen, Germantown, MD, USA) using the QuantiNova SYBR Green kit (Qiagen). The 20 µL reaction mixtures contained 0.7 µM of each primer, 10 µL of QuantiNova SYBR Green master mix, 5 µL of DNA diluted template corresponding to 1 ng of total DNA, and 2.2 µL of RNase-free water. The conditions were as follows: 95 °C for 3 min, followed by 35 cycles of 30 s at 95 °C for denaturation, 30 s at 52 °C for annealing, and 30 s at 60 °C for elongation. Relative quantitation was carried out using all of the bacteria to normalize *S. oneidensis* abundance, and the concentration at day 0 as the calibrator according to the 2^−ΔΔCt^ Method [28].

## 3. Results

### 3.1. Cr(VI) Reduction and Distribution Mass-Balance 

**Figure 1:**
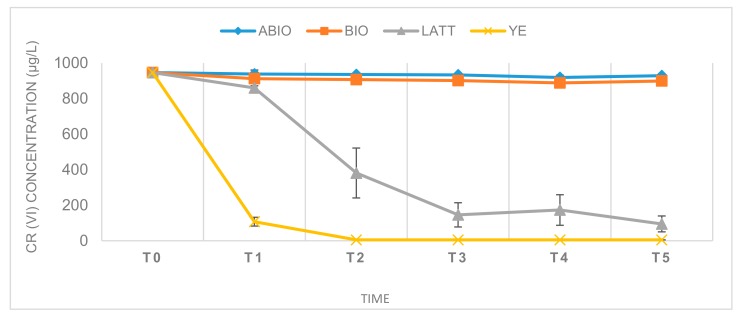
The variation, in time, of observed chromium concentrations in all of the experimental conditions (ABIO, BIO, LATT, and YE) at different times of incubation (0, 4, 7, 11, 14, and 28 days).

Reactors were monitored over a period of about four weeks (28 days). Their kinetic profiles are shown in Figure 1. The yeast extract amendment provided the best reduction of Cr(VI), reaching concentrations below 100 µg/L just four days’ after incubation (T1), and Cr(VI) values under the limit of quantification (5 µg/L) within one week of the experiment (T2).

**Figure 2:**
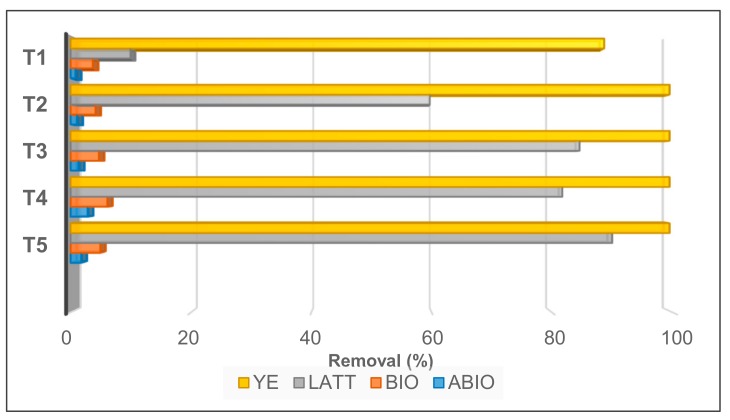
Percentage of Cr(VI) removal observed during different times of incubation (T1, T2, T3, T4, and T5) in microcosm tests setup in different experimental conditions (YE, LATT, BIO, and ABIO).

LATT resulted in a slower Cr(VI) decontamination with respect to YE, achieving a 90% contaminant reduction after 11 days (T3; Figure 2). On the contrary, a minimal decrease of the pollutant was observed in the soil and water not sterilized (BIO), and a not significative decrement in the ABIO microcosms, showing—at the end of the experiment (T5)—Cr(VI) reductions equal to 4.75% and 1.57%, respectively (Figure 2).

**Figure 3:**
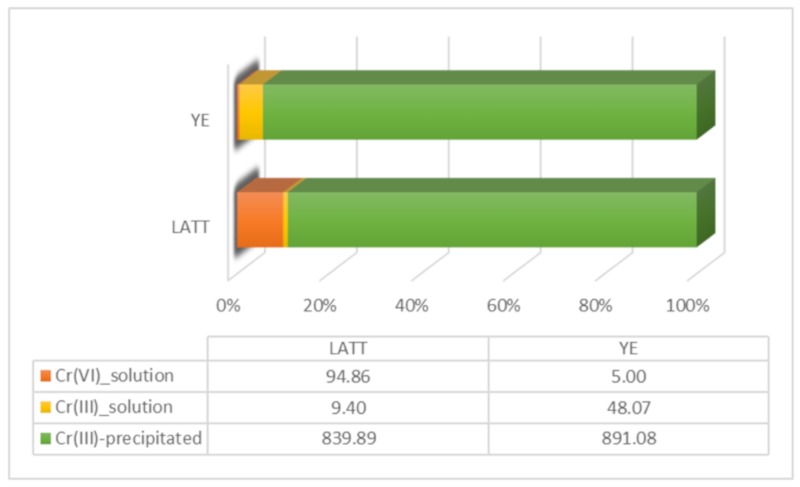
Distribution of Cr fractions in YE and LATT microcosms at the end of the experiment (T5, 28 days). The quantitative data are expressed as µg/L.

Moreover, the ICP-MS chemical analyses revealed a low percentage of Cr(III) in the supernatant solution measured at the end of the experiment (T5) in both the LATT and YE biological microcosms, with a consequent precipitation of Cr(III) products in the solid matrix (Figure 3). In this regard, the presence of black precipitates in the solid phase of the amended microcosms was registered at T5 (28 days); this was consistent in both the YE and LATT reactors.

### 3.2. Control Tests 

**Figure 4:**
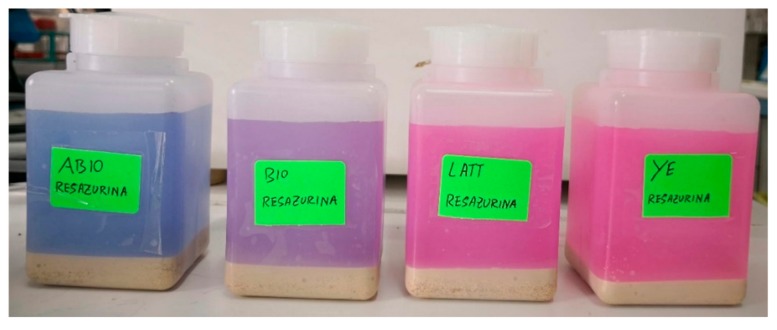
Resazurin control reactors at T2 (seven days of incubation).

The analyses of the control reactors added with resazurin evidenced some differences among the experimental conditions (ABIO, BIO, LATT, and YE). The YE reactors added with resazurin were the first to show an evident change in the colour of the aqueous phase with respect to the initial coloration, suggesting that the redox potential passed from positive to negative values, already at a few days after incubation (T1, four days). Also, the LATT and the BIO resazurin controls, after one week of incubation (T2), revealed a more slight shift in coloration from blue to pink than that detected in the YE ones, indicating that a change in the electrochemical potential was occurring (Figure 4). On the contrary, no change in the ABIO control was observed at all during the experiment, indicating that no anaerobic condition occurred.

The second line of control tests, setup with autoclaved matrices and amendments (LATT control and YE control), revealed a percentage of Cr(VI) removal much lower than that observed in the not autoclaved microcosms (LATT or YE; Figure 1). Specifically, after four days of incubation, the Cr(VI) removal in the LATT control microcosms was about half of that observed in the LATT reactors (4.79% vs 9.98%, respectively), as well as the Cr(VI) removal in the YE control microcosms was equal to one tenth of that evidenced in the YE tests (7.69% vs. 88.65%, respectively).

### 3.3. The Amendments’ Effect on Microbial Abundance 

**Figure 5:**
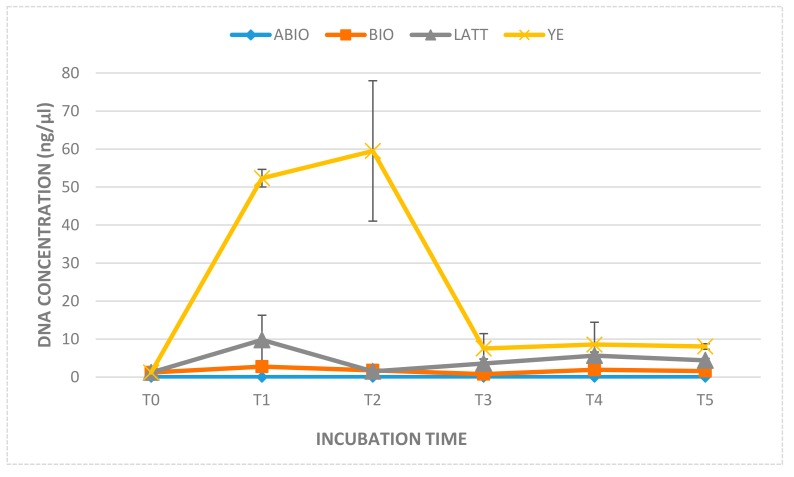
Concentration of genomic DNA extracted from the aqueous phase of each microcosms at different times of investigation (T1, T2, T3, T4, and T5).

The amendments’ effect on microbial abundance was estimated by measuring the amount of the total DNA extracted. The YE microcosms showed the highest amount of DNA at seven days (T2), but in the following times of the analyses, a strong DNA decrement was measured; meanwhile, the LATT series showed a weak improvement trend with raising (T1, T3, and T4) and decreasing values (T2 and T5), as shown in Figure 5. In the BIO reactors, the total amount of DNA remained the same compared to the initial value. No detectable amount of DNA (less than 2 ng for 20 µL, corresponding to the maximum loadable volume of extract) was extracted from the ABIO microcosms.

### 3.4. PCR Assays 

**Figure 6:**
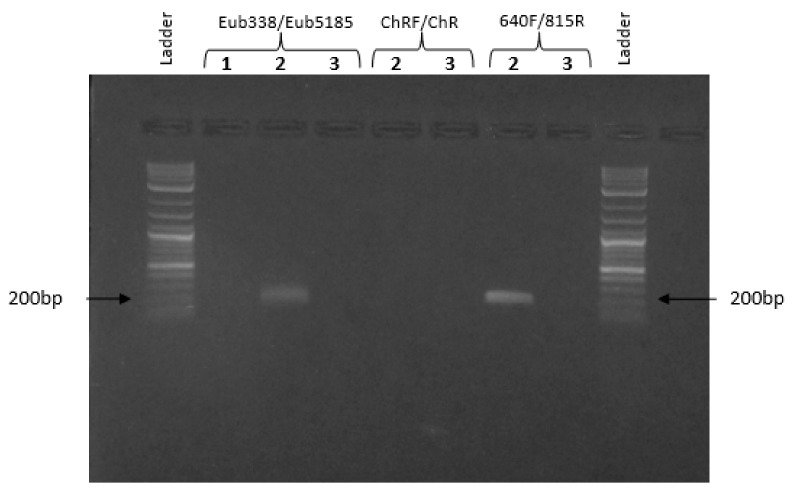
PCR products on 1.5% agarose gel. A 0.1–1 kb ladder was used as the DNA size marker. (**1**) limestone, (**2**) groundwater, and (**3**) no template control.

No PCR products were observed in the limestone after amplification using the Eub338/Eub518 primer set, suggesting that the presence of microorganisms in this matrix was extremely low; meanwhile, the bacterial presence was detectable in the groundwater employed to set up microcosms (amplicon length 200 bp). For this reason, a downstream qPCR application was conducted only on the aqueous phase of each microcosms at the different times of investigation. No evidence for the intracellular chromate reductase (ChR gene) was found in the groundwater, instead, PCR products of 195 bp corresponding to *Shewanella oneidensis* were observed, revealing the natural presence of this microorganism, capable of extracellular Cr(VI) reduction in groundwater (Figure 6).

### 3.5. Assessment of Shewanella Oneidensis in Amended Biological Microcosm

**Figure 7:**
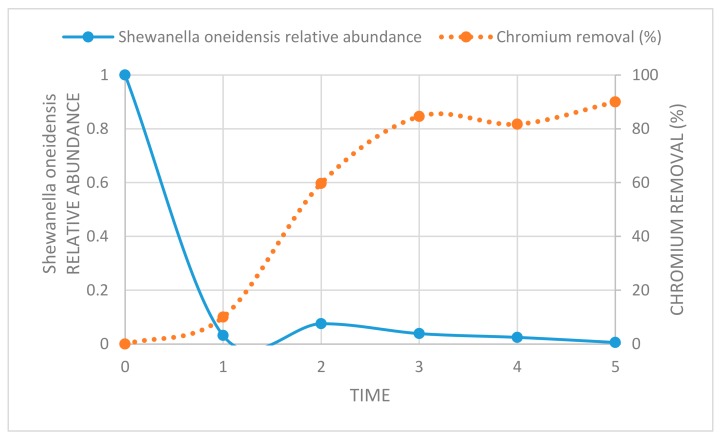
*Shewanella oneidensis* relative abundance, calculated using the concentration at T0 as the calibrator, and normalized using all of the bacteria (continuous blue line) and the chromium removal % (dotted orange line) at different times of investigation: (**0**) 0, (**1**) 4, (**2**) 7, (**3**) 11, (**4**) 14, and (**5**) 28 days.

Focusing on the aqueous phase of the LATT biological microcosms, it is possible to note that, although the amount of total DNA increased four days after the microcosms’ set up, the *Shewanella oneidensis* relative abundance decreased drastically, and this trend persisted during the experiment until 28 days (T5 in Figure 7).

Regarding the aqueous phase of YE microcosms, the *Shewanella oneidensis* relative abundance decreased sharply at time T2 (seven days, data not shown).

## 4. Discussion

The mitigation of the toxic effects of Cr(VI) by reduction to Cr(III) is of great interest for the understanding of the possible bioremediation processes usable for recovering contaminated groundwater. The overall result showed that the enrichment of microcosms by lactate or yeast extract led to a rapid decrease in the Cr(VI) concentration, thus enhancing the biological reduction of this pollutant. In fact, both these amendments provide energy substances useful to support the natural microbial communities of the contaminated aqueous phase in microcosms, favouring their abundance, as evidenced by the concentration of genomic DNA extracted already after days of incubation (T1, Figure 5). At the same time, the control tests setup with autoclaved matrices (limestone and groundwater) and amendments, revealed a scarce degree of Cr(VI) reduction, in particular, in the LATT microcosm (4.79%), significantly lower than that registered in the not sterilized microcosms; in these ones, the highest Cr(VI) removal percentages were reached in the amended reactors, suggesting that the hexavalent chromium removal was mainly “biological”; in this sense, the role of both of the amendments was substantially to stimulate the ability of the autochthonous microbial populations involved in the reductive Cr(VI) process, providing them a carbon source useful for their growth. This was particularly evident in the YE microcosms, where the curve of the extracted DNA concentration reached the highest value at seven days (T2; Figure 5), when all of the Cr(VI) has been reduced, and then it gradually decreases following the exhaustion of nutrients in the aqueous phase.

A non-significative reduction was registered in the ABIO microcosms, in which the Cr(VI) removal percentage was 1.61% on average at the different incubation times (T1 to T5). Cause of the absence of microbial communities in this line of microcosms, the scarce reduction of Cr(VI) is more probably ascribable to the presence of reducing agents naturally occurring in the aqueous phase employed for reactors’ setting up. According to several authors [29,30], some chemical compounds in water, such as organic substances; hydrogen sulphide; sulfur; iron sulphide; ammonium; and nitrite V^2+^-, Fe^2+^-, and Fe(II)-containing minerals, can have a role in reducing the pollutant into Cr(III); therefore, the light hexavalent chromium decrement measured in ABIO microcosms could be detected as a consequence of the occurrence of these substances in the starting matrix. Further analyses will be carried out in the upcoming experiments to identify and quantify these compounds, and thus to understand their role in the chemical reduction of Cr(VI).

The results, relative to the molecular analyses, lead to the hypothesis that an intracellular Cr(VI) reduction pathway—mediated by the cytoplasmic chromate reductase examined in this work—does not occur in the laboratory microcosms (BIO, LATT, and YE). On the contrary, an extracellular reduction pathway seems to take place in the amended microcosms (LATT and YE), as evidenced by the presence of the chromium precipitates (Figure 3) detected in the solid matrix (limestone), according to Gong et al. [31] and Ksheminska et al. [32], and also, by the absence of Cr(VI) and (III) in the aqueous solutions of reactors at the end of the experiment (28 days). Therefore, the Cr(VI) removal could be caused by membrane-associated enzymes, such as reductase, as demonstrated in the research works of Mangaiyarkarasi et al. [33] and Long et al. [34].

Among the bacteria capable of extracellular chromate reduction, *Shewanella oneidensis* does not seem to play a significant role in the bioreduction of Cr(VI) in the experimental conditions realized in this work, because its relative abundance was extremely low, even under 100 µg/L of Cr(VI) (Figure 7). This result was in line with previous studies, in which *Shewanella oneidensis* was highly susceptible to growth inhibition by Cr(VI), even at 15 µg/L [35,36]. In this work, the process of Cr(VI) reduction in all of the experimental conditions (BIO, LATT, and YE) of laboratory microcosms occurred in the absence of oxygen, as confirmed by the resazurin control tests, and according to Cheung and Gu [37], and Qian et al. [38].

**Figure 8:**
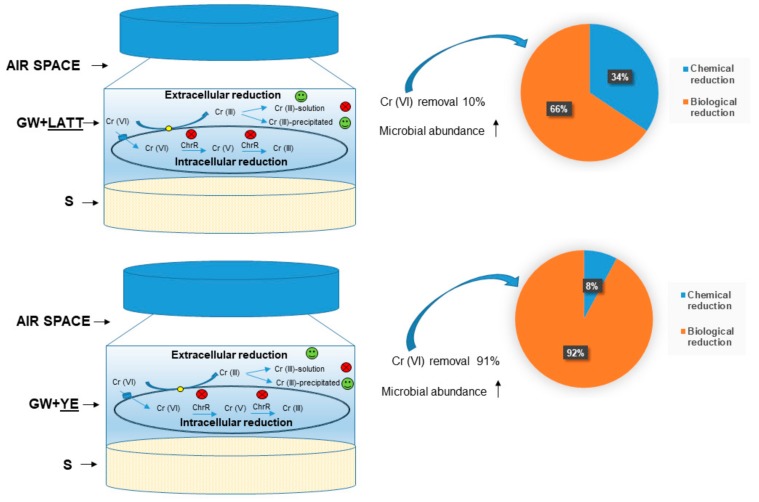
Scheme of the probable mechanisms of Cr (VI) reduction occurred after four days of incubation in the LATT and YE laboratory microcosms. GW—groundwater; S—limestone.

By combining the chemical analyses with molecular investigations, and comparing the resulted data with those from previous batch-type experiments [4,20,39], it has been possible to hypothesize an elucidating scheme of the most probable Cr(VI) reduction mechanism that occurred in LATT and YE, already after four days of incubation (Figure 8). In both experimental conditions, a Cr(VI) removal was observed, reaching 10% in the microcosms amended with LATT and about 90% in the microcosms amended with YE. The extracellular route of Cr(VI) reduction should be preferred to the intracellular one, with the formation of Cr(III) compounds mostly precipitated in the solid phase. In LATT microcosms, the Cr(VI) reduction observed was for 66% biological via autochthonous microorganisms, and for 34% chemical; while in the YE ones, the Cr(VI) reduction observed was mainly biological (92%), ascribable to the activation of autochthonous microbial communities due to the addition of this amendment.

## 5. Conclusions

The overall results presented in this work show the key role of amendments (lactate or yeast extract) in enhancing the biological reduction of hexavalent chromium in the contaminated aqueous phase of laboratory microcosms.

The existence of a natural microbial community is a fundamental condition for an adequate reaction to the various chemical substances that can pollute an ecosystem. Only if the amount and the toxicity of the xenobiotics do not inhibit growth and activity of natural microorganisms, the restoration from pollution is possible.

The use of laboratory microcosms, set up by using bacterial communities collected from natural water or soil, allows for assessing the microbial capabilities to transform contaminants in both these environmental matrices, and then to reduce their toxicity. The knowledge of the presence of autochthonous microbial communities with a natural remediation capacity can be effective for developing biological site-specific recovery strategies for the natural attenuation of contaminated environmental matrices (water and soil). Also, it can be useful for customizing the addition of amendments that promote microbial activity. Furthermore, the application of the specifically identified bacterial strains could enhance the bioremediation capability. In light of this, further investigations will be carried out at a laboratory scale, by using Cr(VI) contaminated groundwater in an industrial area of Southern Italy, in order to (i) evaluate the kinetics of Cr(VI) decontamination at different concentrations, in the presence and absence of specific amendments; (ii) identify the most suitable microbial species for the bio-reduction process of Cr(VI) by Next Generation Sequencing; and (iii) identify appropriate protocols for the biostimulation of bacterial strains in bioremediation treatments. Moreover, spectroscopic analyses, such as Fourier transform infrared (FT-IR) and X-ray photoelectron spectra (XPS), will be performed and combined with chemical and molecular investigations, with the aim of defining a comprehensive mechanism of the hexavalent chromium bio-reduction.

## Figures and Tables

**Table 1:** Experimental conditions for bottle microcosms. ABIO—soil and water sterilized; BIO—soil and water not sterilized; LATT—with the addition of lactate; YE—with the addition of yeast extract; S—limestone; GW—groundwater.

Experimental Condition	Composition	Properties
**ABIO**	S + GW + sodium azide (1 g/L)	Autoclaved matrices with sodium azide
**BIO**	S + GW	Not autoclaved matrices
**LATT**	S + GW + lactate (3 mM)	Not autoclaved matrices amended with lactate
**YE**	S + GW + yeast extract (200 mg/L)	Not autoclaved matrices amended with yeast extract

**Table 2:** Primers used for PCR amplifications in this work.

Method Used	Sequence Primers	Amplicon Length	Genome	Reference
**Eub338**	ACTCCTACGGGAGGCAGCAG	200 bp	16S rRNA gene All of the bacteria	[25]
**Eub518**	ATTACCGCGGCTGCTGG			
**ChR**	CGTACCCTGATCAATCACTT	268 bp	Chromate reductase gene	[26]
**ChRF**	TCACGCCGGAATATAACTAC			
**640F**	RACTAGAGTCTTGTAGAGG	195 bp	V3–V4 *Shewanella oneidensis*	[27]
**815R**	AAGDYACCAAAYTCCGAGTA			
